# Terpenoid Compositions of Resins from *Callitris* Species (*Cupressaceae*)

**DOI:** 10.3390/molecules23123384

**Published:** 2018-12-19

**Authors:** Bernd R. T. Simoneit, Robert E. Cox, Daniel R. Oros, Angelika Otto

**Affiliations:** 1 Department of Chemistry, College of Science, Oregon State University, Corvallis, OR 97331, USA; 2 Consultant, 24 Francis Street, Blackburn, Victoria 3130, Australia; cox.robert448@gmail.com; 3 Consultant, 72 Marina Lakes Drive, Richmond, CA 94804, USA; 4 Forschungsinstitut Senckenberg, Sektion Paläeobotanik, Senckenberganlage 25, 60325 Frankfurt/Main, Germany; simonellit@yahoo.de

**Keywords:** *Callitroideae*, diterpenoids, GC-MS, standards

## Abstract

The environmental fate of conifer resins and their natural product compounds as mixtures is of importance for source, alteration, and transport studies. The compound compositions of resins of the common *Callitris* species (*Cupressaceae*) based on gas chromatography-mass spectrometry have not been reported. Results show that diterpenoids were the most abundant components and callitrisic acid was present in the resin extracts of all *Callitris* species analyzed. Significant amounts of 4-*epi*-pimaric and sandaracopimaric acids, with lesser communic, ozic, and lambertianic acids, were also in the mixtures. Phenolic diterpenoids, for example, ferruginol, hinokiol, were found in trace quantities in some samples. Thus, callitrisic acid and 4-*epi*-pimaric acid are the characteristic diterpenoids of *Callitris* species that are amenable to molecular biomarker analyses in geological or environmental applications.

## 1. Introduction

Natural products from plants (e.g., terpenoids of conifer resins) are preserved directly or as derivatives (diagenetic products) in the contemporary and fossil geological environments. When extracted and characterized, they are used as molecular biomarkers in organic geochemistry, paleontology, forensics, archeology, and environmental chemistry for source identification [1,2,3,4,5,6,7,8,9,10,11,12,13,14]. The application of gas chromatography-mass spectrometry (GC-MS) in the analysis of natural product mixtures extracted from plants for compound characterization can also be of utility for rapid screening in pharmacological studies [15].

The Coniferae are known as important source plants for resins and are comprised of *Araucariaceae* (3 genera), *Cupressaceae* (27 genera), *Pinaceae* (11 genera), *Podocarpaceae* (18 genera), *Taxaceae* (6 genera), and *Sciadopityaceae* (1 genus) [16]. Here we focus on the *Cupressaceae*, specifically the genus *Callitris* with 19 species, because there is a paucity on the characterization of their natural terpenoid compositions [17].

Dehydroabietic acid (abieta-8,11,13-trien-18-oic acid) is the most commonly encountered and stable molecular biomarker from conifer resins [1]. However, its epimer, callitrisic acid (abieta-8,11,13-trien-19-oic acid), has not been reported for contemporary sedimentary environments. Nevertheless, callitrisic acid and degradation products, such as 16,17-bisnorcallitrisic acid, and 9,10-*seco*-callitrisic acids, are found in certain ambers or their pyrolysates [12,18,19].

Callitrisic acid was isolated from *Callitris columellaris* wood and its structure was determined by correlation with known compounds and synthesis [20]. Additional resin acids, including 7-oxocallitrisic acid, were also reported [21]. Callitrisic acid has a restricted distribution in the extant plant kingdom, mainly in *Callitris* species and isolated reports for *Juniperus*, *Calceolaria*, *Rabdosia*, and *Illicium* species, the latter three belonging to Angiosperms [15,22,23,24]. The only other natural products reported from *Callitris* species are sesquiterpenoids and lignans. The sesquiterpenoids are comprised of mainly callitrisin, columellarin, and isomers in wood of *C. columellaris* [25,26,27]. The lignans podophyllotoxin and deoxypodophyllotoxin have been detected in *C. drummondii* and *C. columellaris*, respectively [28,29,30]. In this study only *C. preissii* contains numerous known and novel lignans in the total resin extract. They are a complex mixture, including *seco*-lariciresinols, lariciresinols, pinoresinols, and matairesinol with many syringyl moieties, and their mass spectra as the derivatized compounds with interpretations have been published [31].

Here we report a survey by GC-MS of the dominant resin components of nine common *Callitris* species, and an assessment of the presence of callitrisic acid in resins of closely related and other conifers. 

## 2. Experimental

### 2.1. Samples

The samples were collected as hardened, freshly bled resins from the stems of various *Callitris* species (*Cupressaceae*, subfamily *Callitroideae*). In the case of species with no obvious resin, a branchlet was sampled and air dried prior to extraction. The samples and their source locations are given in Table 1. The resins of other conifers were sampled and analyzed in the same manner.

### 2.2. Extraction and Gas Chromatography-Mass Spectrometry

The resin samples and dried branchlets were crushed and sonicated three times with dichloromethane:methanol (DCM:MeOH, 3:1, *v*/*v*) for 15 min. The total extracts were combined, filtered, and concentrated with a rotary evaporator and then with nitrogen blow down (to typically 1–3 mL). Aliquots (50 µL) of the total extracts were converted to trimethylsilyl (TMS) derivatives by reaction with N,O-bis(trimethylsilyl)trifluoroactamide (BSTFA) and pyridine for 3 h at 70 °C. Prior to GC-MS analysis, the excess silylating reagent was evaporated under a dry nitrogen stream and the sample mixture was dissolved in an equivalent volume of *n*-hexane. Other aliquots (50 µL in DCM:MeOH, 1:1 *v*/*v*) were treated with trimethylsilyldiazomethane (20 µL, 2 M in *n*-hexane, Sigma-Aldrich, St. Louis, MO, USA) to methylate carboxylic acids prior to analysis. This reaction proceeded at room temperature within 30 min, after which the excess reagent was removed with acetic acid (glacial grade), followed by blow down with nitrogen and dissolution in *n*-hexane.

GC-MS analyses of the underivatized and derivatized extracts were carried out using an Agilent model 6890 GC coupled to an Agilent model 5973 quadrupole MSD. GC-MS data were acquired with the associated Chemstation software. Identifications of compounds were based on comparisons with standards, literature mass spectra, Wiley 275 library data, and interpretation of mass spectrometric fragmentation patterns for unknown compounds. The mass spectra of novel compounds and their derivatives (methyl esters or TMS esters/ethers), with the basic fragmentation patterns, are also presented. The relative abundance of each significant compound was calculated using its peak area in the respective total ion current (TIC) trace and assuming the same response factor.

## 3. Results and Discussion

The sesqui- and diterpenoids identified in the resins of *Callitris* sp. and their relative abundances are listed in Table 2. The Kovats GC retention indices of the natural products or their derivatives relative to *n*-alkanes are given on the respective mass spectra [32].

### 3.1. Resin Compositions

**Figure 1:**
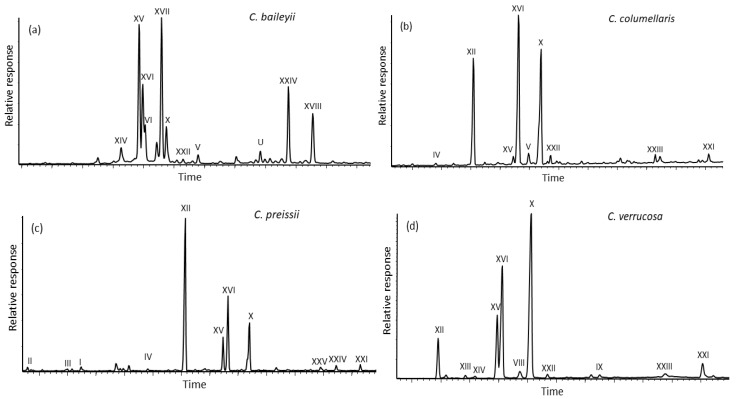
Examples of total ion current (TIC) traces for total extracts of *Callitris* species resins: (**a**) *C. baileyii* analyzed as the methylated and silylated extract, (**b**) *C. columellaris* analyzed as the methylated extract, (**c**) *C. preissii* analyzed as the methylated extract (the major lignans are not shown), and (**d**) *C. verrucosa* analyzed as the methylated extract. Roman numerals refer to the compounds in Table 2. U = unknown.

The diterpenoid compositions of the samples are quite diverse, especially with regards to callitrisic acid (X, the chemical structures are given in Appendix A and follow the sequence in Table 2). Some examples of total resin compositions are shown in Figure 1. Callitrisic acid (X) is the dominant compound in resins from *C. intratropica*, *C. macleayana*, *C. rhomboides*, and *C. verrucosa*, a trace component in resin of *C. oblonga*, and minor in the other samples. Dehydroabietic acid (XI) is a trace component only in resin of *C. muelleri* and 16,17-bisnorcallitrisic acid is not detectable. Sandaracopimaric acid (XVI), 4-*epi*-pimaric acid (XII), communic acids (XIII-XV), and 12E-ozic acid (XVII) are the secondary major components (Table 2). Various hydroxycallitrisic acids (XXIII–XXVI), lambertianic acid (XXII), and 7-oxocallitrisic acid (XXI) are also significant in some of the resins. Callitrisol (V), ferruginol (VI), and sandaracopimara-8(14),15-dien-3β-ol (VII) are minor hydroxylated components in some samples. In addition, *C. preissii* resin contains dominant lignans, as already reported [31]. Three sesquiterpenoids, i.e., callitrisin (I), columellarin (II), and dihydrocolumellarin (III), are present here only in resin of *C. preissii* (Figure 1c). These were reported before in heartwood of *C. columellaris* [25,26], but not detected in our resin sample.

### 3.2. Mass Spectrometry

**Figure 2:**
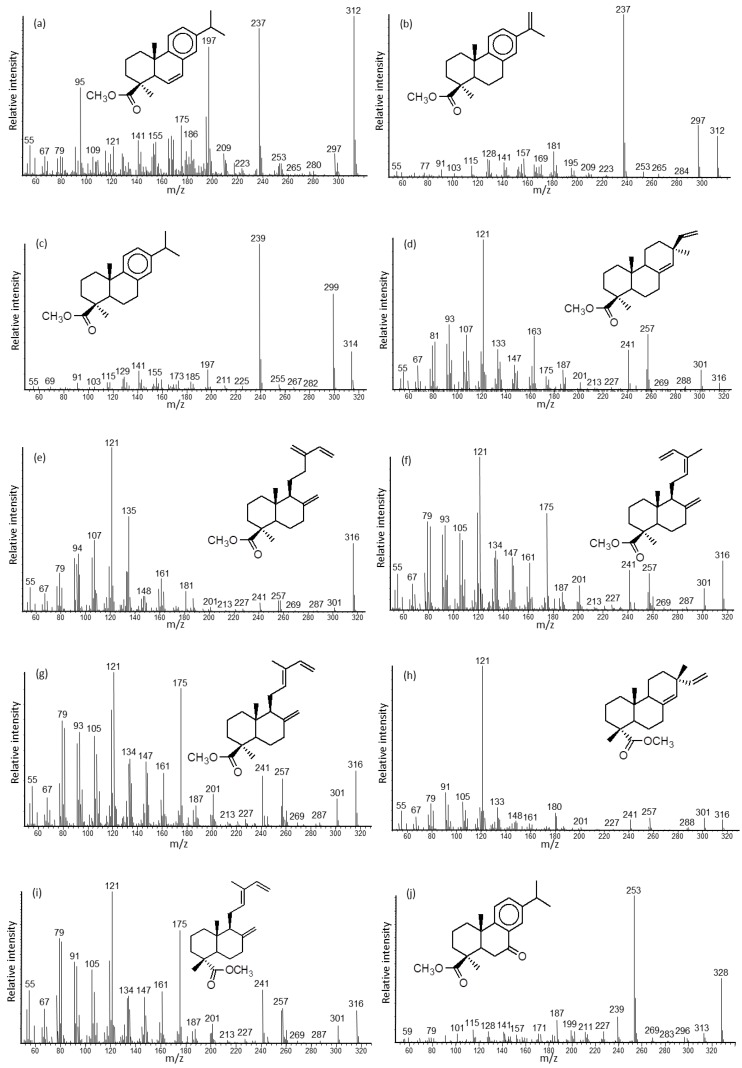

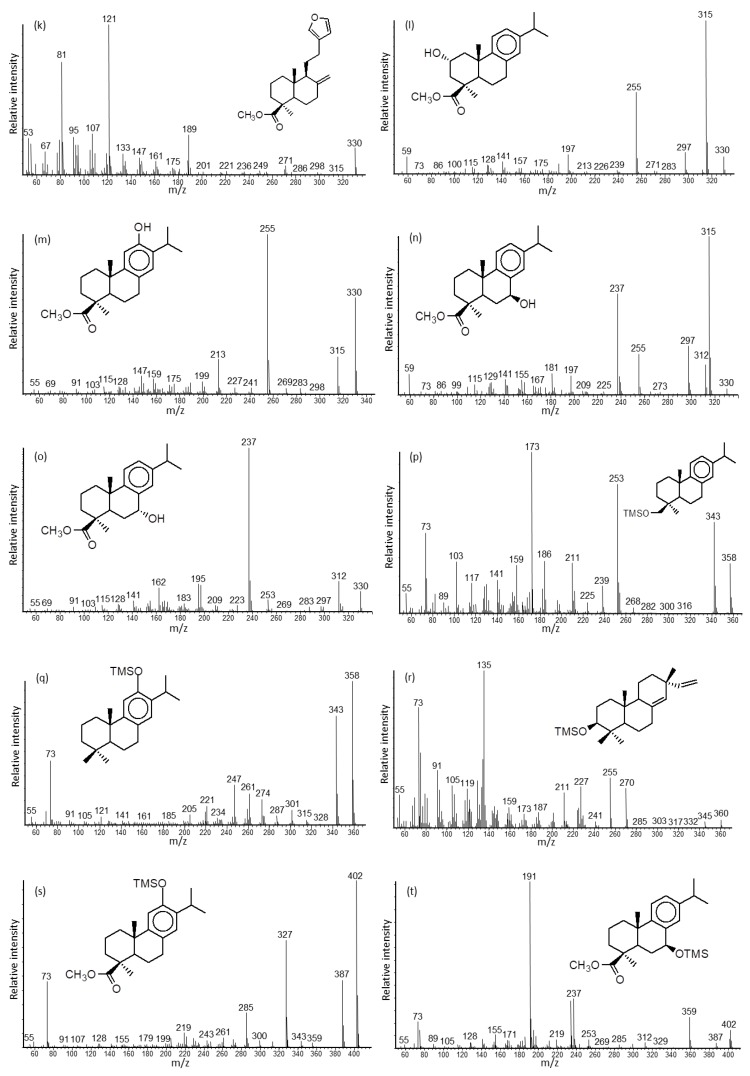

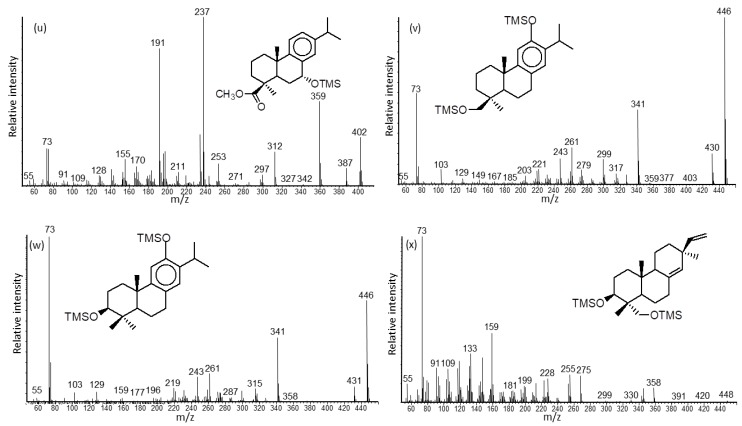
Mass spectra of the terpenoids listed in Table 2, analyzed as the natural products, methylated and/or silylated derivatives: (**a**) methyl abieta-6,8,11,13-tetraene-19-oate (VIII), (**b**) methyl abieta-8,11,13,15-tetraen-19-oate (IX, [33]), (**c**) methyl callitrisate (methyl abieta-8,11,13-trien-19-oate, X), (**d**) methyl 4-*epi*-pimarate (XII, [34]), (**e**) methyl *iso*-communate (XIII), (**f**) methyl 12Z-communate (XIV), (**g**) methyl 12E-communate (XV), (**h**) methyl sandaracopimarate (XVI), (**i**) methyl 12E-ozate (XVII), (**j**) methyl 7-oxocallitrisate (XXI, [21]), (**k**) methyl lambertianate (XXII), (**l**) methyl 2α-hydroxycallitrisate (XXIII), (**m**) methyl 12-hydroxycallitrisate (XXIV, [35]), (**n**) methyl 7β-hydroxycallitrisate (XXV), (**o**) methyl 7α-hydroxycallitrisate (XXVI, [23]), (**p**) callitrisol-TMS (V), (**q**) ferruginol-TMS (VI), (**r**) sandaracopimaradien-3β-ol (VII), (**s**) methyl 12-hydroxycallitrisate-TMS (XXIV), (**t**) methyl 7β-hydroxycallitrisate-TMS (XXV), (**u**) methyl 7α-hydroxycallitrisate-TMS (XXVI), (**v**) 12-hydroxycallitrisol-diTMS (XVIII), (**w**) hinokiol-diTMS (XIX), and (**x**) 3β,18-dihydroxypimaradiene-diTMS (XX).

The mass spectra of the compounds in Table 2, analyzed as the free and derivatized products, are shown in Figure 2. Additional mass spectra of related and derivatized natural products are collected and discussed in the Supplemental Materials.

Callitrisic acid (X) was easily distinguished from its isomers, i.e., dehydroabietic (XI), 5β-callitrisic, 5β-dehydroabietic, and veadeiroic (cleistantha-8,11,13-trien-19-oic, [36]) acids, based on the GC retention index and mass spectrum (Figure 2c versus Figure S1r–u, Supplemental Material). The methyl esters had the best resolution and stability for analysis by GC-MS, versus their trimethylsilyl esters (Figure S1aa–cc). The key ion was *m*/*z* 239 with intense molecular (M^+^) ion at *m*/*z* 314 (30) and M-CH_3_ ion at *m*/*z* 299 (65), compared to methyl dehydroabietate with M^+^ at *m*/*z* 314 (10) and M-CH_3_ at *m*/*z* 299 (11). Traceamounts of Δ^6^- and Δ^15^-callitrisic acids (VIII, XI, respectively) were also found (Figure 2a,b), and were identified by correlation with the standard isomers of methyl Δ^6^- and Δ^15^-dehydroabietates and veadeiroates (Figure S1n–q).

The presence of 4-*epi*-pimaric acid (XII) is of interest. The identification was based on its early GC elution and the same mass spectrum as that of pimaric acid standard (Figure 2d and Figure S1dd), coupled with a literature report [34]. The mass spectra of the communic acids (XIII-XV) and sandaracopimaric acid (XVI) match those of the respective standards (Figure 2e–h and Figure S1ee–hh). The communic acids have been characterized for resin from *C. columellaris* [21]. Ozic acid (XVII, 4-*epi*-communic acid, assumed 12E-isomer) was a dominant component in two samples, and its mass spectra (Figure 2i and Figure S1ii) were interpreted by comparison with literature data [37,38] and the GC retention indices versus those of the communic acids. Lambertianic acid (XXII) is a major component in most samples and its mass spectra (Figure 2k and Figure S1nn) were interpreted by comparison with a surrogate standard from resin of *Pinus lambertiana* [39]. 7-Oxocallitrisic acid (XXI) is a significant oxidation product in many samples and its mass spectra (Figure 2j and Figure S1mm) were interpreted by comparison with standard 7-oxodehydroabietic acid and GC retention index.

The mass spectra of the sesquiterpenoids callitrisin (I), columellarin (II) and dihydrocolumellarin (III) were inferred from previous listings [25,26] (Figure S1a–c). The mass spectrum of dehydroabietane (IV) has been presented before [40] (Figure S1d), ferruginol (VI) matches with the standard (Figure 2q and Figure S1f), sandaracopimaradien-3β-ol (VII) correlates with literature data (Figure 2r and Figure S1g), and hinokiol (XIX) correlates with the surrogate standard from resin of *Chamaecyparis obtusa* (Figure 2w and Figure S1l).

### 3.3. Environmental and Geological Implications

**Figure 3:**
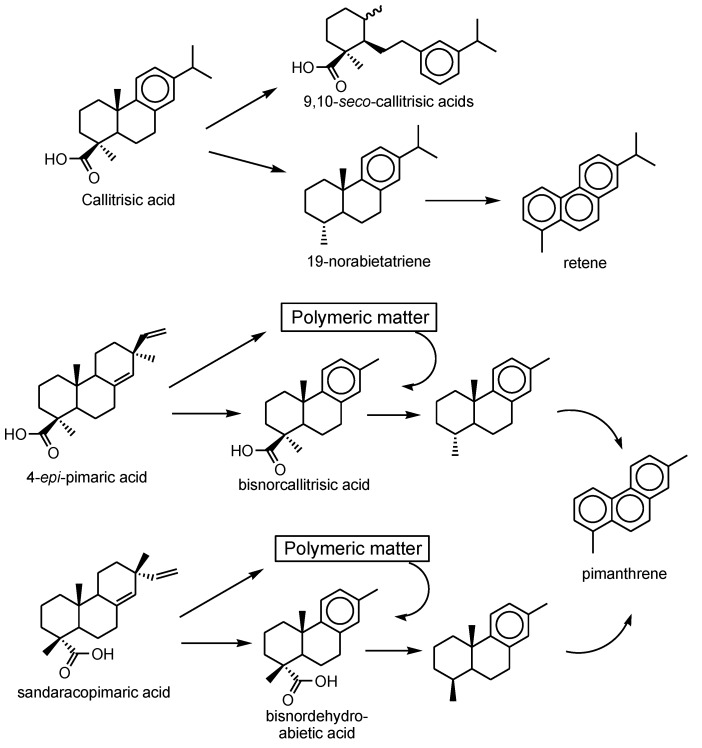
Diagenetic products from callitrisic, 4-*epi*-pimaric and sandaracopimaric acids.

The environmental fate of conifer resins and their natural product compounds as mixtures is of importance for source, alteration and transport studies [10,41,42,43]. The precursor–product relationship for diterpenoids based on the abietane and pimarane skeletons has been presented by numerous authors [1,42,44,45]. Thus, callitrisic acid, 4-*epi*-pimaric acid, ferruginol and lambertianic acid of the *Callitris* resins were proposed as the main environmental tracers. Over geological timespans, the fate of the communic and ozic acids is oxidation and incorporation into macromolecular polymers. The diagenetic fate of callitrisic acid is decarboxylation with subsequent aromatization, analogous as dehydroabietic acid, to the same hydrocarbons, i.e., dehydroabietin (18- or 19-norabieta-8,11,13-triene) and retene (Figure 3). Also, 4-*epi*-pimaric acid may aromatize to 15,16-bisnorcallitrisic acid by loss of C_2_H_6_, or become incorporated into polymeric matter across the C-15 to C-16 double bond with subsequent release as the same diagenetic product (Figure 3). Bisnordehydroabietic acid may be derived by the same route from sandaracopimaric acid (Figure 3). These products are readily observed in pyrolysates of some ambers [18]. 

**Figure 4:**
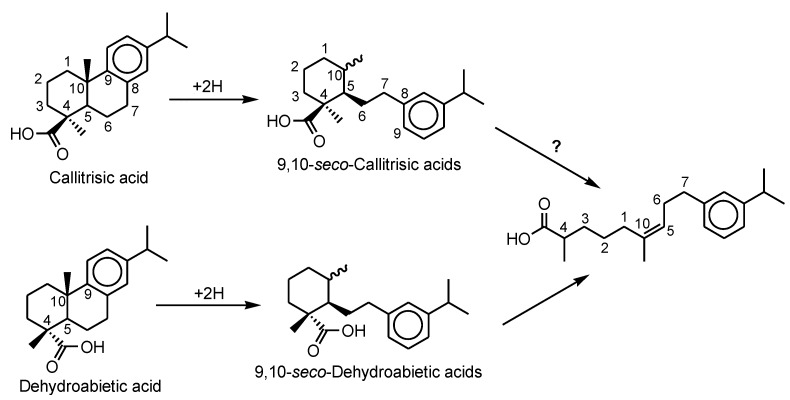
Ring-opening isomerization of callitrisic and dehydroabietic acids.

The unknown factor is whether callitrisic acid can also isomerize to dehydroabietic acid in fossil resins. Dehydroabietic acid is generally the dominant compound in total extracts of certain ambers and fossil resins, with minor or trace amounts of callitrisic acid [12,18,19,46]. We also found the *seco*-derivatives of both callitrisic and dehydroabietic acids in some amber extracts and commonly in aged pine resins (see the mass spectra in the Supplemental Materials). The pine resins contained dehydroabietic acid, 10α(H)- and 10β(H)-9,10-*seco*-dehydroabietic acids, and 4,5,9,10-bis-*seco*-dehydroabietic acid [2,6-dimethyl-9-(3′-(2-methylethyl)phenyl)non-2-enoic acid]; whereas the ambers contained both sets of *seco*-derivatives, but the bis-*seco*-compound was not found. We propose that the 10α(H)- and 10β(H)-9,10-*seco*-callitrisic acids may also proceed to the 4,5,9,10-bis-*seco*-derivative (Figure 4). Furthermore, we speculate if these reactions are reversible in amber, then ring reclosures may lead to epimerization at C-4.

We found no callitrisic acid in the closely related species (e.g., *Diselma archeri*, *Fitzroya cupressoides*, *Tetraclinis articulata*, and *Austrocedrus chilensis* [47,48]). We were not able to detect any callitrisic acid in resins of *Juniperus chinensis* and *J. phoenicea*, as reported before [23,49,50]. However, we did find 4-*epi*-abietic and 4-*epi*-pimaric acids in the juniper resins we analyzed. They could dehydrogenate to the aromatic derivatives upon weathering, as for example the rapid oxidation of abietic acid to dehydroabietic acid. Macrofossils of *Callitris* species are rare [51], so further work on the preservation of the major resin tracer components by direct or extract analyses remains for the future.

## 4. Conclusions

Callitrisic acid was found in resin extracts of all *Callitris* species analyzed here. Significant amounts of 4-*epi*-pimaric and sandaracopimaric acids, with lesser communic, ozic, and lambertianic acids, were also in the mixtures. Phenolic diterpenoids, e.g., ferruginol, hinokiol, were found in trace amounts in some samples. Therefore, callitrisic acid and 4-*epi*-pimaric acid are the characteristic diterpenoids of *Callitris* species for molecular biomarker analyses in geological or environmental applications. Furthermore, callitrisic acid has not been found in closely related *Cupressaceae* species, although it is present in some Angiosperms.

## Figures and Tables

**Table 1:** *Callitris* species sampled.

Botanical Name	Common Name	Sample Type	Sample Location	Number of Analyses
*Callitris baileyii*	Bayley’s cypress pine	Resin	RBG, Melbourne, AU	3
*Callitris columellaris, syn. C. glauca*	White cypress pine	Resin	RBG, Melbourne, AU	4
*Callitris intratropica*	Blue cypress	Resin	RBG, Sydney, AU	3
*Callitris macleayana*	Stringybark cypress	Resin	RBG, Sydney, AU	1
*Callitris muelleri*	Illiwara/Bush cypress	Twig	RBG, Melbourne, AU	3
*Callitris oblonga*	Pigmy cypress pine	Twig	RBG, Melbourne, AU	1
*Callitris preissii*	Rottnest Island pine	Resin	RBG, Melbourne, AU	8
*Callitris rhomboidea*	Port Jackson/Oyster Bay pine	Resin	RBG, Melbourne, AU	3
*Callitris verrucosa*	Mallee pine	Resin	RBG, Melbourne, AU	1

AU = Australia; RBG = Royal Botanical Garden.

**Table 2:** Relative concentrations of the major terpenoids in the *Callitris* species resins.

Number	Compound	Composition	MW	Kovats Index ^a^	ID ^b^	*C. Bail.*	*C. Colum.*	*C. Intratr.*	*C. Maclea.*	*C. Muell.*	*C. Oblonga*	*C. Preissii*	*C. Rhomb.*	*C. Verruc.*
I	Callitrisin	C_15_H_20_O_2_	232	1916	L							10		
II	Columellarin	C_15_H_20_O_2_	232	1925	L							24		
III	Dihydrocolumellarin	C_15_H_22_O_2_	234	1875	L							6		
IV	Dehydroabietane	C_20_H_30_	270	2084	S		1						0.5	
V	Callitrisol	C_20_H_30_O	286	2152	I	5	2	4				8		
VI	Ferruginol	C_20_H_30_O	286	2289	S	20	0.3	0.2	0.6	19		1.3		
VII	Sandaracopimaradien-3β-ol	C_20_H_32_O	288	2110	L		7			90				
VIII	∆^6^-Callitrisic acid	C_20_H_26_O_2_	298	2318	I			8					8	4
IX	∆^15^-Callitrisic acid	C_20_H_26_O_2_	298	2436	I								2	2
X	Callitrisic acid	C_20_H_28_O_2_	300	2325	S	24	75	94	100	16	2.8	31	100	100
XI	Dehydroabietic acid	C_20_H_28_O_2_	300	2358	S				2	1.5	1.2			
XII	4-*epi*-Pimaric acid	C_20_H_30_O_2_	302	2166	I		69	26			60	100	20	23
XIII	*iso*-Communic acid	C_20_H_30_O_2_	302	2225	L		1		11				3	2
XIV	12Z-Communic acid	C_20_H_30_O_2_	302	2263	L	6		9	15			1	2	1
XV	12E-Communic acid	C_20_H_30_O_2_	302	2268	L	90	5	40	54		100	21	38	38
XVI	Sandaracopimaric acid	C_20_H_30_O_2_	302	2275	S	34	100	100	29	95		48	15	69
XVII	Ozic acid (4-*epi*-communic acid)	C_20_H_30_O_2_	302	2317	L	100				100				
XVIII	19-Hydroxyferruginol	C_20_H_30_O_2_	302	2517 *	S	34								
XIX	Hinokiol	C_20_H_30_O_2_	302	2537 *	L					49				
XX	3β,18-Dihydroxypimara-8(14),15-diene	C_20_H_32_O_2_	304	2457 *	L					90				
XXI	7-Oxocallitrisic acid	C_20_H_26_O_3_	314	2531	I		5	5	9			5	16	12
XXII	Lambertianic acid	C_20_H_28_O_3_	316	2355	L	3	5	3	4	12	4		2	3
XXIII	2α-Hydroxycallitrisic acid	C_20_H_28_O_3_	316	2386 *	I		6		4				8	2
XXIV	12-Hydroxycallitrisic acid	C_20_H_28_O_3_	316	2526 *	I	52								
XXV	7β-Hydroxycallitrisic acid	C_20_H_28_O_3_	316	2407 *	I								8	
XXVI	7α-Hydroxycallitrisic acid	C_20_H_28_O_3_	316	2390 *	I								10	

^a^ As free compounds or methyl esters, * = TMS derivative; ^b^ S = standard, L = literature citation, I = interpretation of MS fragmentation pattern.

## Supplemental Material

Additional mass spectra of related and derivatized natural products are collected and discussed in the Supplemental Material section available with this paper.

## Appendix A

Chemical structures cited.
