# Supplementary material for: Terpenoid Compositions of Resins from Callitris Species (Cupressaceae)
Source: Molecules. 2018 Dec 19;23(12):3384. doi: 10.3390/molecules23123384 (PMC6321214; doi:10.3390/molecules23123384)
Supplement: Supplementary file 1 [file molecules-23-03384-s001.pdf]

Supplemental Materials

for

Terpenoid compositions of resins from *Callitris* species (Cupressaceae)

Bernd R.T. Simoneit[1], Robert E. Cox[2], Daniel R. Oros[3], Angelika Otto[4]

[1] Department of Chemistry, College of Science, Oregon State University, Corvallis, OR 97331, U.S.A.

[2] 24 Francis Street, Blackburn, Victoria 3130, Australia

[3] 72 Marina Lakes Drive, Richmond, CA 94804, U.S.A.

[4] Forschungsinstitut Senckenberg, Sektion Paläeobotanik, Senckenberganlage 25, 60325 Frankfurt/Main, Germany

Contents

Figure S1. Mass spectra of derivatives and related or isomeric terpenoids.

Figure S1. Mass spectra of derivatives and related or isomeric terpenoids:

(a) Callitrisin (I),

(b) columellarin (II),

(c) dihydrocolumellarin (III),

(d) dehydroabietane (IV, standard),

(e) callitrisol (V),

(f) ferruginol (VI, standard),

(g) sandaracopimara-8(14),15-dien-3β-ol (VII),

(h) callitrisic acid (X, standard),

(i) dehydroabietic acid (XI, standard),

(j) veadeiroic acid (standard),

(k) 19-hydroxyferruginol (XVIII, standard),

(l) hinokiol (XIX),

(m) 3β,18-dihydroxypimara-8(14),15-diene (XX),

(n) methyl abieta-6,8,11,13-tetraen-18-oate (standard),

(o) methyl cleistantha-6,8,11,13-tetraen-19-oate (standard),

(p) methyl abieta-8,11,13,15-tetraen-18-oate,

(q) methyl cleistantha-8,11,13,15-tetraen-19-oate (standard),

(r) methyl dehydroabietate (XI, standard),

(s) methyl veadeiroate (standard),

(t) methyl 5β-callitrisate (standard),

(u) methyl 5β-dehydroabietate (standard),

(v) methyl 10β(H)-9,10-*seco*-callitrisate,

(w) methyl 10α(H)-9,10-*seco*-callitrisate,

(x) methyl 10β(H)-9,10-*seco*-dehydroabietate,

(y) methyl 10α(H)-9,10-*seco*-dehydroabietate,

(z) methyl 4,5,9,10-bis-*seco*-dehydroabietate,

(aa) callitrisic acid-TMS (X, standard),

(bb) dehydroabietic acid-TMS (XI, standard),

(cc) veadeiroic acid-TMS (standard),

(dd) 4-*epi*-pimaric acid-TMS (XII),

(ee) *iso*-communic acid-TMS (XIII),

(ff) 12Z-communic acid-TMS (XIV),

(gg) 12E-communic acid-TMS (XV),

(hh) sandaracopimaric acid-TMS (XVI, standard),

(ii) ozic acid-TMS (XVIII),

(jj) 10β(H)-9,10-*seco*-dehydroabietic acid-TMS,

(kk) 4,5,9,10-bis-*seco*-dehydroabietic acid-TMS,

(ll) 10α(H)-9,10-*seco*-dehydroabietic acid-TMS,

(mm) 7-oxodehydroabietic acid-TMS (standard),

(nn) lambertianic acid-TMS (XXII, standard).

Note - structures shown on mass spectra and listed above are a reference standard, or are tentative based on a literature citation and interpretation of the fragmentation pattern with consideration of the relative GC retention index.

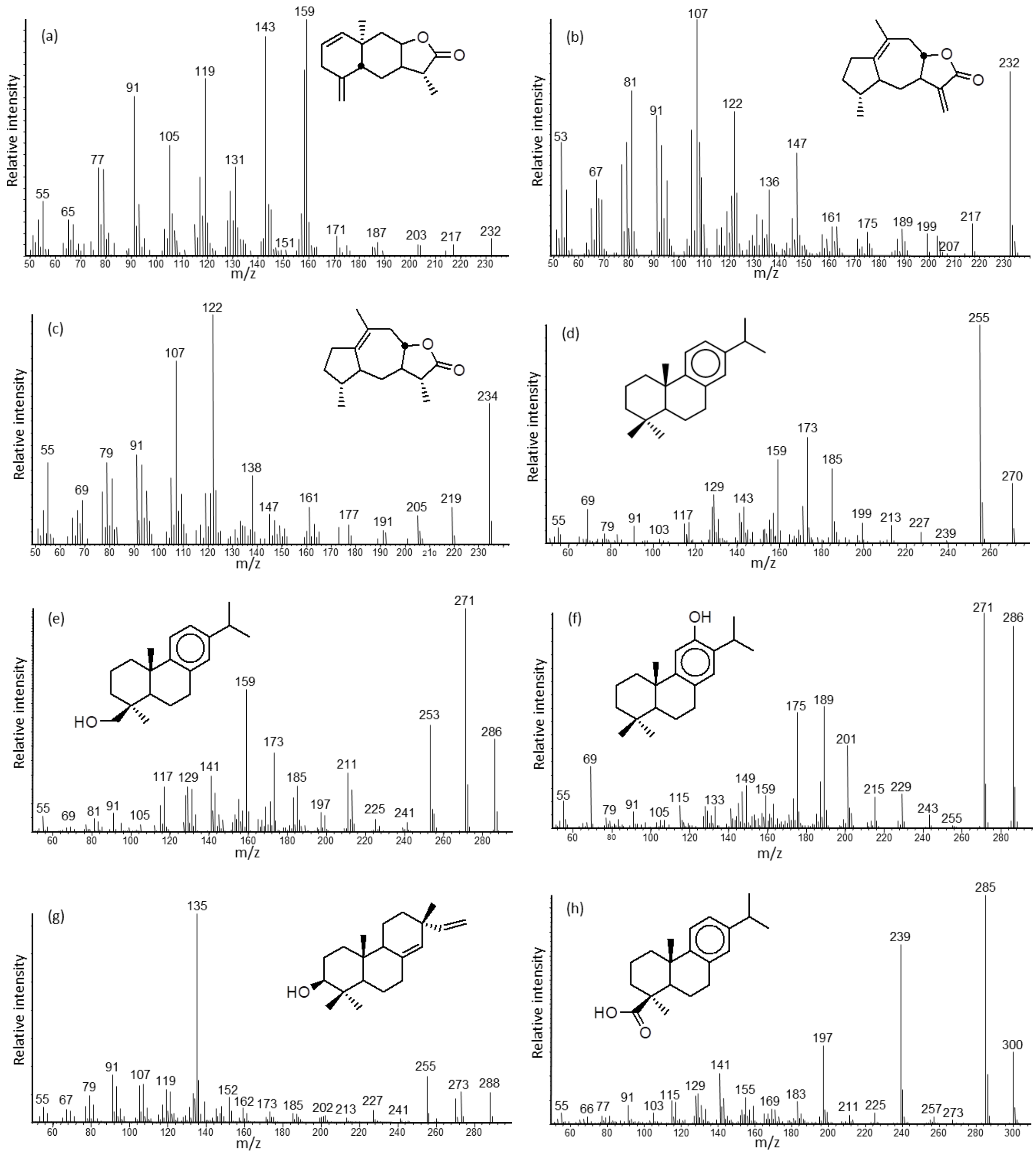
(a)
Relative intensity
55 65 77 91 105 119 131 143 151 159 171 187 203 217 232
m/z
(b)
Relative intensity
53 67 81 91 107 122 136 147 161 175 189 199 207 217 232
m/z
(c)
Relative intensity
55 69 79 91 107 122 138 147 161 177 191 205 219 234
m/z
(d)
Relative intensity
55 69 79 91 103 117 129 143 159 173 185 199 213 227 239 255 270
m/z
(e)
Relative intensity
HO
55 69 81 91 105 117 129 141 159 173 185 197 211 225 241 253 271 286
m/z
(f)
OH
Relative intensity
55 69 79 91 105 115 133 149 159 175 189 201 215 229 243 255 271 286
m/z
(g)
Relative intensity
HO
55 67 79 91 107 119 135 152 162 173 185 202 213 227 241 255 273 288
m/z
(h)
Relative intensity
HO
O
55 66 77 91 103 115 129 141 155 169 183 197 211 225 239 257 273 285 300
m/z

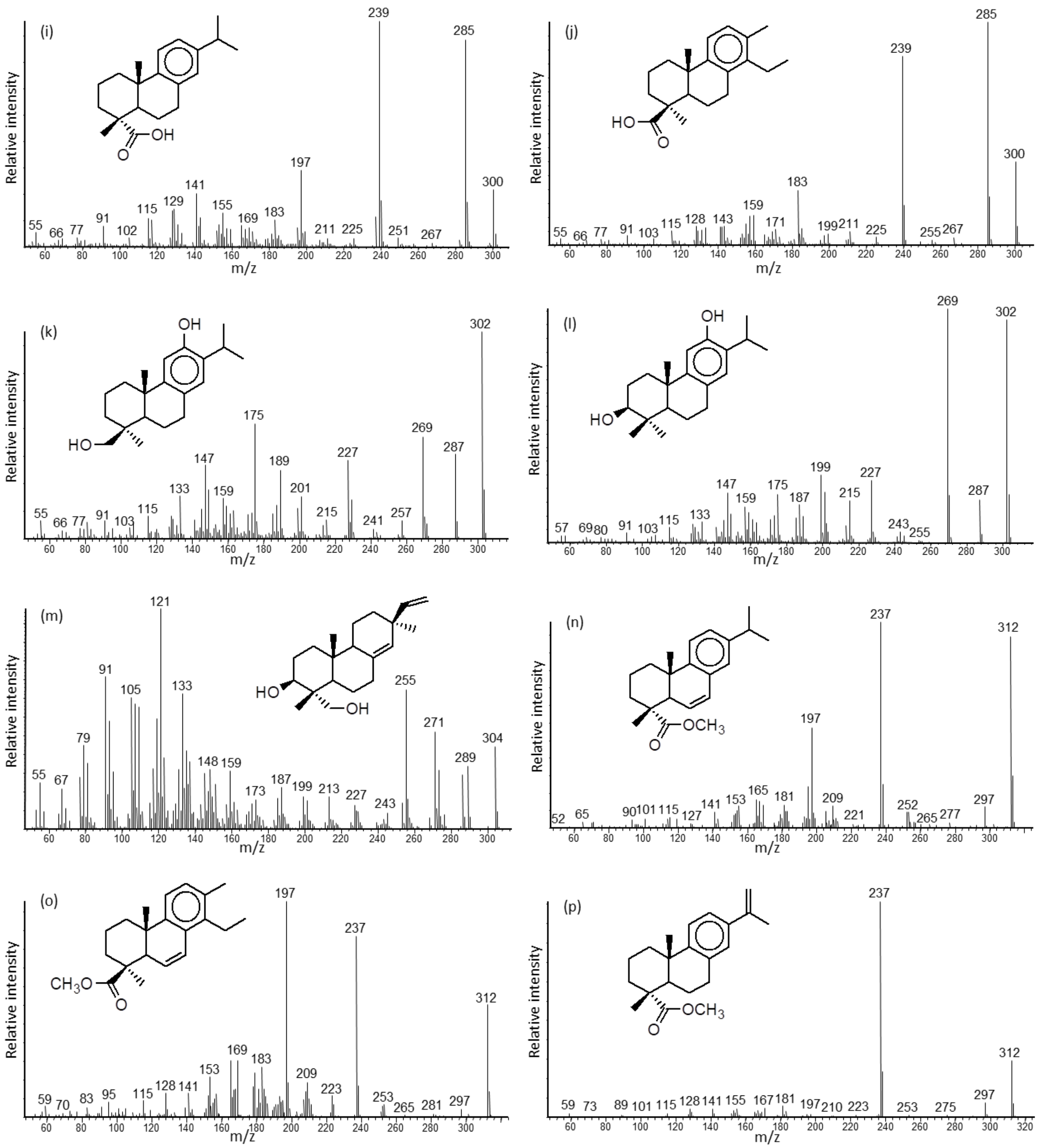
(i)
OH
O
55 66 77 91 102 115 129 141 155 169 183 197 211 225 239 251 267 285 300
(j)
HO
O
55 66 77 91 103 115 128 143 159 171 183 199 211 225 239 255 267 285 300
(k)
OH
HO
55 66 77 91 103 115 133 147 159 175 189 201 215 227 241 257 269 287 302
(l)
OH
HO
57 69 80 91 103 115 133 147 159 175 187 199 215 227 243 255 269 287 302
(m)
HO
OH
55 67 79 91 105 121 133 148 159 173 187 199 213 227 243 255 271 289 304
(n)
OCH3
O
52 65 90 101 115 127 141 153 165 181 197 209 221 237 252 265 277 297 312
(o)
CH3O
O
59 70 83 95 115 128 141 153 169 183 197 209 223 237 253 265 281 297 312
(p)
OCH3
O
59 73 89 101 115 128 141 155 167 181 197 210 223 237 253 275 297 312
Relative intensity
m/z

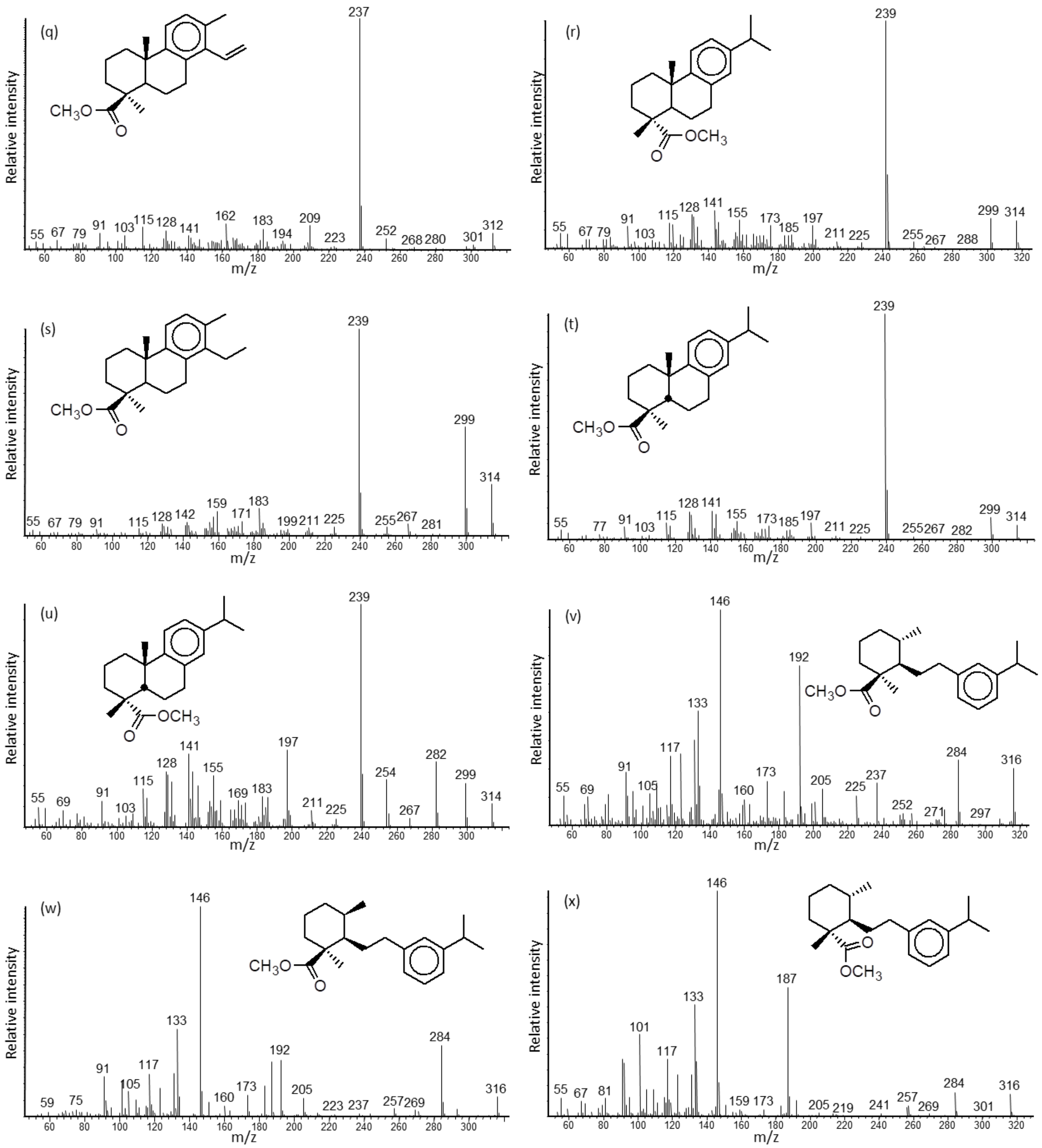
(q)
CH3O
O
237
55 67 79 91 103 115 128 141 162 183 194 209 223 252 268 280 301 312
(r)
OCH3
O
239
55 67 79 91 103 115 128 141 155 173 185 197 211 225 255 267 288 299 314
(s)
CH3O
O
239
299
314
55 67 79 91 115 128 142 159 171 183 199 211 225 255 267 281
(t)
CH3O
O
239
55 77 91 103 115 128 141 155 173 185 197 211 225 255 267 282 299 314
(u)
OCH3
O
239
55 69 91 103 115 128 141 155 169 183 197 211 225 254 267 282 299 314
(v)
CH3O
O
146
192
133
55 69 91 105 117 160 173 205 225 237 252 271 284 297 316
(w)
CH3O
O
146
133
192
284
59 75 91 105 117 160 173 205 223 237 257 269 316
(x)
O
OCH3
146
187
133
101
55 67 81 117 159 173 205 219 241 257 269 284 301 316
Relative intensity
m/z

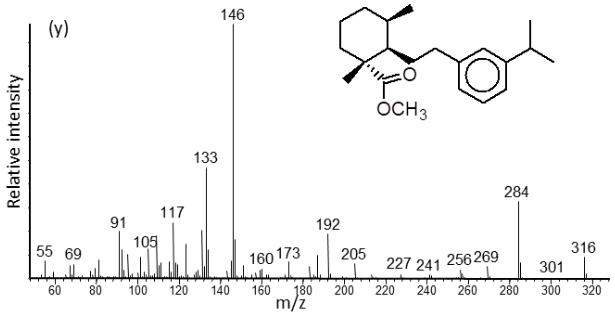
(y)
Relative intensity
m/z
OCH3
55 69 91 105 117 133 146 160 173 192 205 227 241 256 269 284 301 316

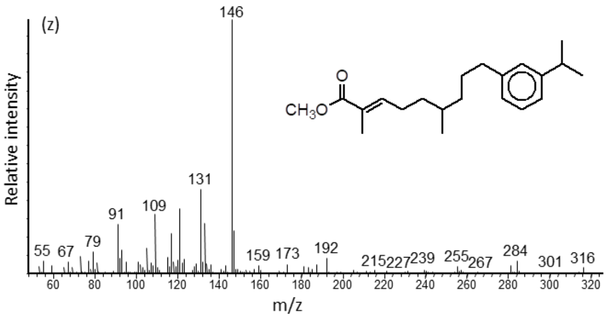
(z)
Relative intensity
m/z
CH3O
55 67 79 91 109 131 146 159 173 192 215 227 239 255 267 284 301 316

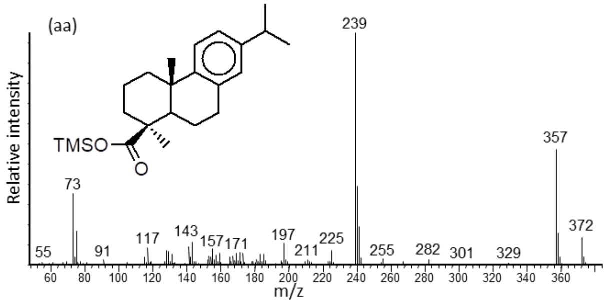
(aa)
Relative intensity
m/z
TMSO
55 73 91 117 143 157 171 197 211 225 239 255 282 301 329 357 372

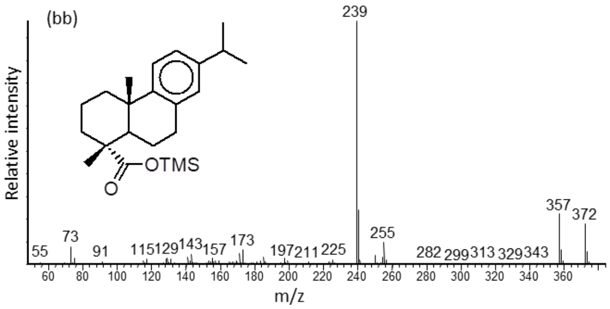
(bb)
Relative intensity
m/z
OTMS
55 73 91 115 129 143 157 173 197 211 225 239 255 282 299 313 329 343 357 372

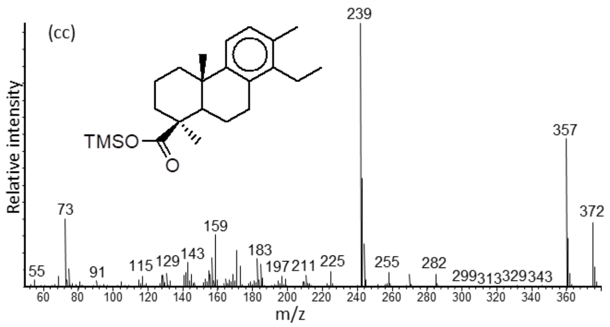
(cc)
Relative intensity
m/z
TMSO
55 73 91 115 129 143 159 183 197 211 225 239 255 282 299 313 329 343 357 372

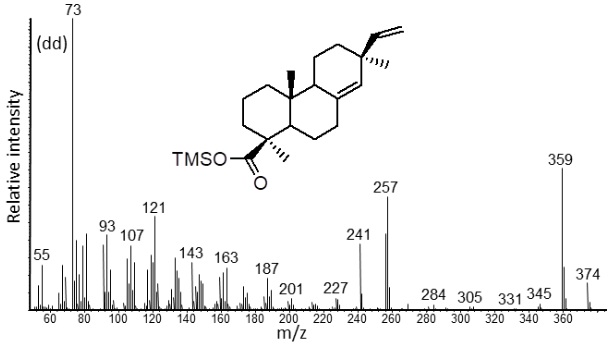
(dd)
Relative intensity
m/z
TMSO
55 73 93 107 121 143 163 187 201 227 241 257 284 305 331 345 359 374

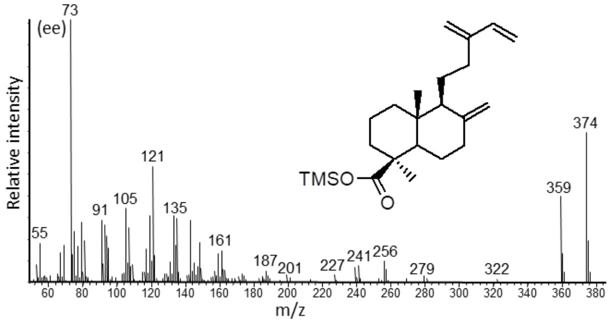
(ee)
Relative intensity
m/z
TMSO
55 73 91 105 121 135 161 187 201 227 241 256 279 322 359 374

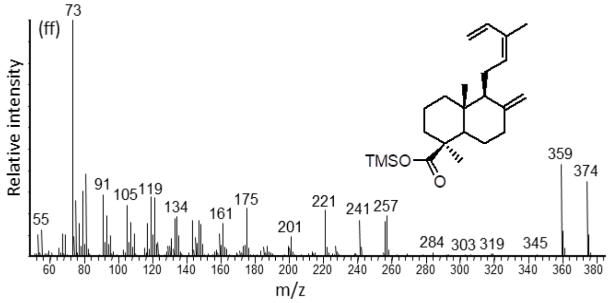
(ff)
Relative intensity
m/z
TMSO
55 73 91 105 119 134 161 175 201 221 241 257 284 303 319 345 359 374

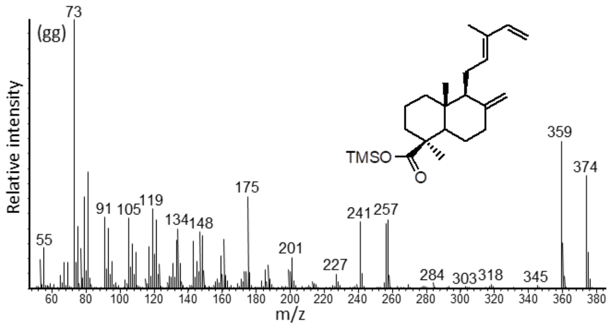
(gg)
Relative intensity
m/z
TMSO
55
73
91 105 119
134 148
175
201
227
241 257
284 303 318
345
359
374
60 80 100 120 140 160 180 200 220 240 260 280 300 320 340 360 380

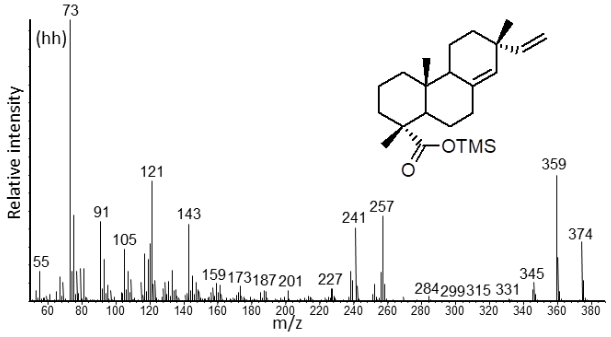
(hh)
Relative intensity
m/z
OTMS
55
73
91
105
121
143
159 173 187 201
227
241 257
284 299 315 331
345
359
374
60 80 100 120 140 160 180 200 220 240 260 280 300 320 340 360 380

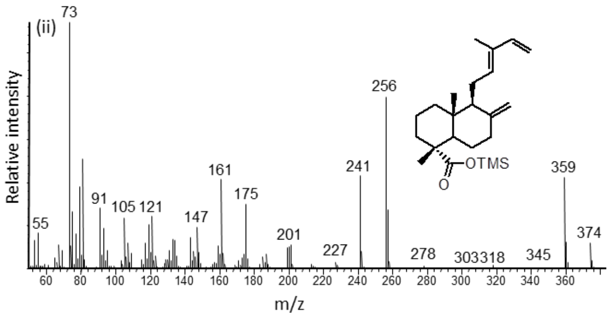
(ii)
Relative intensity
m/z
OTMS
55
73
91 105 121
147
161
175
201
227
241
256
278 303 318
345
359
374
60 80 100 120 140 160 180 200 220 240 260 280 300 320 340 360

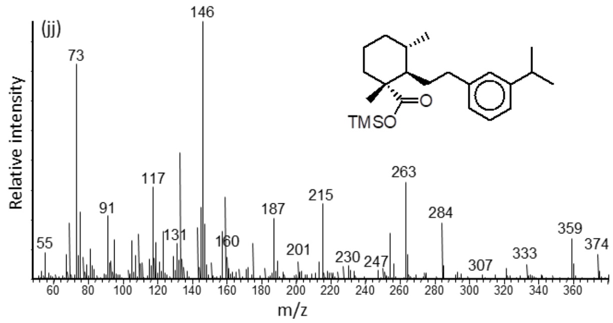
(jj)
Relative intensity
m/z
TMSO
55
73
91
117
131
146
160
187
201
215
230 247
263
284
307
333
359
374
60 80 100 120 140 160 180 200 220 240 260 280 300 320 340 360

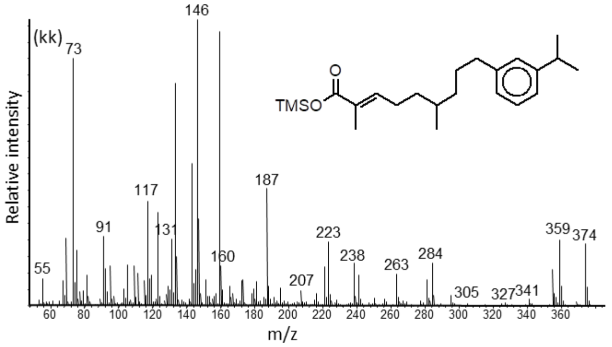
(kk)
Relative intensity
m/z
TMSO
55
73
91
117
131
146
160
187
207
223
238
263
284
305 327 341
359 374
60 80 100 120 140 160 180 200 220 240 260 280 300 320 340 360

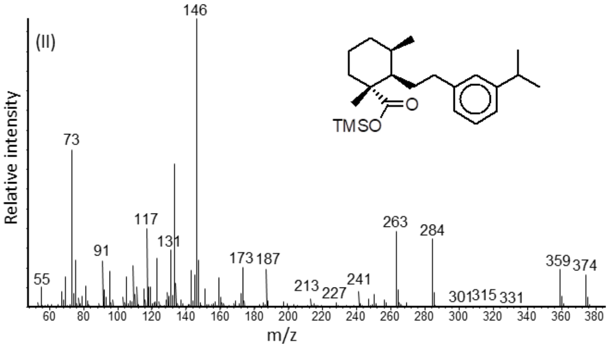
(ll)
Relative intensity
m/z
TMSO
55
73
91
117
131
146
173 187
213 227
241
263
284
301 315 331
359 374
60 80 100 120 140 160 180 200 220 240 260 280 300 320 340 360 380

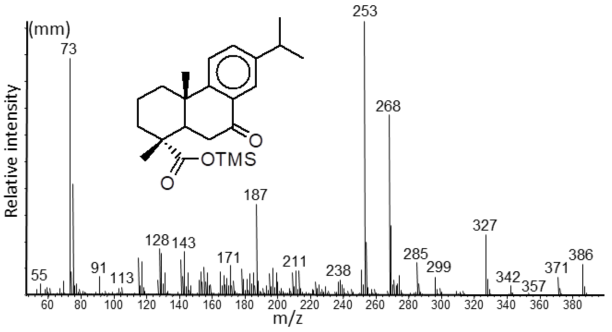
(mm)
Relative intensity
m/z
OTMS
55
73
91 113
128 143
171
187
211
238
253
268
285
299
327
342
357
371
386
60 80 100 120 140 160 180 200 220 240 260 280 300 320 340 360 380

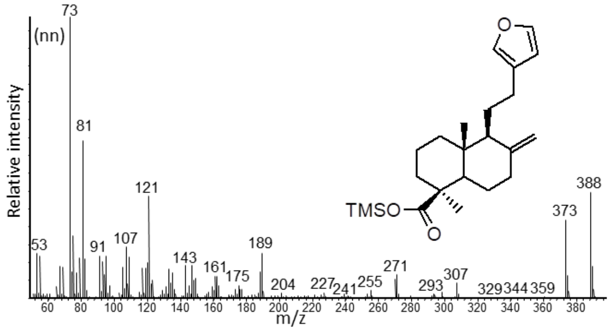
(nn)
Relative intensity
m/z
TMSO
53
73
81
91
107
121
143
161
175
189
204
227
241
255
271
293
307
329 344 359
373
388
60 80 100 120 140 160 180 200 220 240 260 280 300 320 340 360 380
